# Role of triage audit in an ongoing differentiated TB care initiative to reduce deaths in Tamil Nadu, India

**DOI:** 10.5588/pha.25.0015

**Published:** 2025-09-03

**Authors:** A. Jeyakumar, S. Kalaiselvi, D. Nair, R. Vijayaprabha, D. Kabir, J.M. Melfha, T. Bhatnagar, R. Srinivasan, K. Gayathri, K. Boopathi, R.S. Vaman, V. Rajan, S. Shanmugasundaram, A. Frederick, H.D. Shewade

**Affiliations:** ^1^ ICMR-National Institute of Epidemiology, Chennai, India;; ^2^ All India Institute of Medical Sciences (AIIMS), Madurai, India;; ^3^ ICMR-National Institute for Research in Tuberculosis, Chennai, India;; ^4^ District Hospital, Kasaragod, Kasaragod, India;; ^5^ Directorate of Medical and Rural Health Services, Government of Tamil Nadu, Chennai, India.

**Keywords:** tuberculosis, triage audit, severely ill, quality improvement, India, SORT IT

## Abstract

**OBJECTIVE:**

In the ongoing India’s first state-wide differentiated TB care programme in Tamil Nadu (TN-KET), adults diagnosed with drug-sensitive TB at public facilities undergo triage. The adults with severe undernutrition, respiratory insufficiency, or poor performance status are prioritised for comprehensive assessment and inpatient care. Although the programme met triage coverage targets, 11 districts failed to achieve the goal of a 30% reduction in TB death rates. This study compares aggregate triage coverage with actual coverage and evaluates the quality of programme-reported triaging data against an investigator-led audit (repeat assessments in the field) within a few weeks of diagnosis.

**DESIGN:**

An ecological study using routine programme data (April 2022–June 2024) was conducted for the first objective, and a cross-sectional analytical study with primary and secondary data (August 2024–February 2025) was performed for the triage audit.

**RESULTS:**

Among 48,905 adults with drug-sensitive TB notified, the true triage coverage was 84% against the reported triage coverage of 113%. The triage audit showed 35.7% were triage-positive, compared with 27.6% through TB SeWA (Severe TB Web Application). The mean weight and body mass index from the audit were 0.82 kg and 0.63 kg/m^2^ lower than TB SeWA data, and oedema was unassessed in 65% of the adults with TB.

**CONCLUSION:**

The districts need to address inadequate triage coverage and suboptimal quality of triaging.

In 2023, there were 1.3 million estimated deaths globally due to TB. India accounts for the largest number of incident TB (26%) and TB-related deaths (36%) in the world.^1^ Factors such as undernutrition, comorbidities, and alcohol consumption are found to be associated with TB deaths.^2,3^ Triaging for severity followed by appropriate care is recommended as one of the strategies to reduce TB deaths.^4^ Tamil Nadu, the southernmost state in India with a population of around 80 million, notifies ∼88,600 people with TB and reports ∼5,000 TB-related deaths annually.^5^ More than one third of the TB-related deaths are observed within 2 months of diagnosis.^6^

Starting April 2022, Tamil Nadu implemented the *Kasanoi Erappila Thittam* (TN-KET, meaning TB death-free initiative in Tamil), India’s first state-wide differentiated TB care initiative aimed at reducing TB deaths.^7–9^ All adults (≥15 years) with drug-sensitive TB notified from public facilities are triaged at diagnosis for three conditions (five indicators).^6^ The adults identified with very severe undernutrition (body mass index [BMI] ≤ 14 kg/m^2^ or BMI 14.1–16 with bilateral pedal oedema), respiratory insufficiency (respiratory rate > 24/min or oxygen saturation < 94%), or poor performance status (inability to stand without support), called triage-positive, are prioritised for comprehensive assessment and inpatient care.^7–9^ This triage tool has been introduced in routine health systems after successful pilots in two states of India during 2020–2021.^10–12^ Estimates suggest implementing TN-KET could result in a 30% relative reduction in TB death rates.^11^ As of July 2024, the TN-KET quarterly review showed many districts met triage coverage (>90%) and admission rate (>90% among triage-positive) targets, contributing to a reduced TB death rate. However, some districts failed to achieve a 30% relative reduction in deaths despite meeting these targets, and some had death rates exceeding triage positivity rates. Suboptimal triage quality may have led to misclassification, preventing timely inpatient care. In some districts, the number triaged in TB SeWA (Severe TB Web Application, a web-based triage platform) exceeded notifications in Ni-kshay (TB programme management system), suggesting that aggregate-reported triage coverage may not reflect actual coverage among notified persons with TB from public facilities. Triaging coverage based on programme-reported aggregate numbers (89%, the number triaged in TB SeWA divided by the number notified in Ni-kshay) was higher than triaging coverage reported (81%, based on individual tracking between Ni-kshay and TB SeWA). The gap between effective triage coverage and reported triage coverage was only 8% (89% − 81%). However, in 11 districts with no TN-KET effect, this gap was way higher. It was 29% (113% − 84%). TN-KET has documented and addressed quality issues in comprehensive assessment of triage-positive patients at nodal inpatient care facilities.^13^ However, the quality of triaging at the time of diagnosis in the notified facilities has not been explored.

This article describes the triage quality issues identified during the initial phase of quality improvement (triage audit), with the intent of informing the next phase of quality improvement measures.

The specific objectives were to 1) compare the reported triage coverage based on aggregate numbers and true triage coverage based on individual-level tracking from Ni-kshay to TB SeWA and 2) determine the quality of triaging for severe TB in terms of classification, reporting, and measurement errors.

## METHODS

The National Tuberculosis Elimination Programme (NTEP) in Tamil Nadu is implemented through 35 district TB cells, 461 TB units, and 1,984 public TB diagnosis centres. Various TB diagnostic facilities notify TB under the public sector.

TN-KET was implemented across 30 districts of Tamil Nadu, excluding Chennai. Upon diagnosis of drug-sensitive TB, health staff triage patients using a paper-based tool, and senior treatment supervisors transcribe these data into TB SeWA. The platform captures the care cascade from triage results (for triage-negative patients) to discharge (for triage-positive patients), with process indicators available for quarterly review and monitoring. The programme focuses on adults with drug-sensitive TB as triaging offers no operational benefit for drug-resistant TB or paediatric patients. Drug-resistant TB patients undergo routine pre-treatment evaluation, while presumptive paediatric TB patients are referred to a paediatrician.^7–9^ TN-KET is managed by the Tamil Nadu health system under the state TB cell in Chennai, operating without external funding. ICMR-NIE, Chennai, provides technical support, including planning, implementation, monitoring, and operational research, with its team offering mentorship and assistance across all districts. As of July 2024, 11 districts did not meet the goal of a 30% relative reduction in TB death rates. The proportion of triage-positive patients in these districts was 11.4%, not significantly different from 12.8% in the remaining 19 districts (see Table 1). The quality of triaging could be one of the reasons for the differences in TB death rate reduction.

**TABLE 1.** Yearly care cascade in TN-KET, stratified by districts that met/not met the 30% TB death rate reduction goal as of July 2024, Tamil Nadu, India, April 2022–June 2024.

Year	District type	Total triaged, N	Triage-positive, n (%)	Reported TB death rate, %
2022	Poor performing (n = 11)	16,469	2,353 (14.3)	8.2
	Better performing (n = 19)	23,300	2,346 (10.1)	6.1
2023	Poor performing (n = 11)	22,994	2,858 (12.4)	4.3
	Better performing (n = 19)	30,358	3,575 (11.8)	3.0
2024	Poor performing (n = 11)	11,864	1,397 (11.8)	4.7
	Better performing (n = 19)	16,592	2,102 (12.7)	3.8
2022–2024	Poor performing (n = 11)	51,327	6,608 (12.9)	5.4
	Better performing (n = 19)	70,250	8,023 (11.4)	4.0

### Study population and design

Adults (≥15 years) with drug-sensitive TB notified from public health facilities from 11 districts of Tamil Nadu (that did not meet the 30% death reduction goal) were the study population. For the first objective, an ecological study involving routinely collected programme (secondary) data was conducted among adults with TB notified between April 2022 and June 2024. For the second objective, a cross-sectional analytical study involving primary and secondary data was conducted among adults with TB notified between August 2024 and February 2025. The study used purposive sampling based on feasibility and programme needs. Recently diagnosed patients (10 per district) were prioritised for accurate recall and home assessments. High-TB-burden blocks and districts with elevated TB death rates were chosen to boost audit efficiency and data depth. This may limit generalisability due to overrepresentation of high-burden areas and potential selection bias.

### Data collection and sources

**FIGURE 1.**
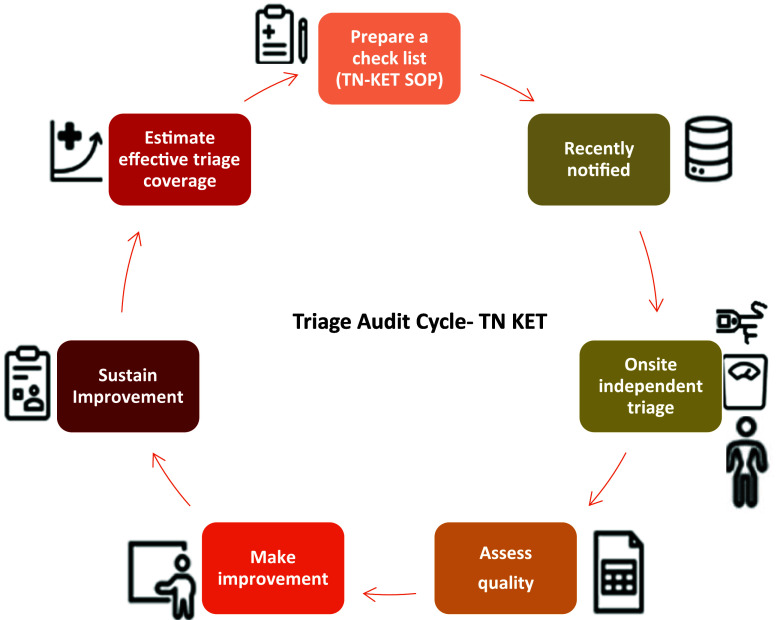
Triage audit processes.

For the first objective, aggregate numbers were extracted (1 March 2025 – date of extraction) from Ni-kshay and TB SeWA for reported triage coverage, and individual-level adults with TB were tracked from Ni-kshay to TB SeWA for true triage coverage (merged using the unique identifier Ni-kshay ID) (see Table 2). The reported triage coverage could be more than 100% as TB SeWA does not have mechanisms to prevent the entry of triage data of drug-resistant and privately notified adults with TB. For the second objective, investigators from the ICMR-NIE TN-KET technical support unit performed the triage audit in the selected districts and blocks. The various steps in the audit cycle are shown in Figure 1. In line with the TN-KET triage tool, a checklist was prepared and used in the field at the residence of an adult with TB.^7^ The onsite audit observations were captured using the mobile-based data capture tool EpiCollect5. The anthropometric measurements, pedal oedema, and oxygen saturation were checked as per the standards prescribed under TN-KET. The weight was measured using a digital scale with an accuracy range of ±100 g. Respiratory assessment was not done under the triage audit. Investigators were of the view that it may not be culturally appropriate to do this at a patient’s residence, especially among women. Under TN-KET, respiratory rate assessment is routinely done in the diagnosing health facility, preferably by the staff nurse, ensuring privacy. This omission could affect comparability with TB SeWA data, where respiratory rate is considered during routine triage and documented accordingly. Therefore, the audit findings on classification error, based solely on undernutrition and performance status indicators, may underestimate the true misclassification rate.

**TABLE 2.** Operational definitions for various assessments done under triage coverage and quality.

Reported triage coverage based on aggregate numbers
Aggregate number of adults with TB notified during a specific period and entered in TB SeWA (triaged) divided by district wise aggregate number of adults with TB notified in Ni-kshay during the same specific notification period
True triage coverage based on individual-level tracking
Number of adults with TB triaged and entered in TB SeWA (triaged) among the adults with TB notified in Ni-kshay during the specific notification period
Classification error
If the inference of the extent of BMI (very severe and severe under nutrition) and triage-positive differs significantly between the audit team and TB SeWA
Reporting error
If the patient reports that pedal oedema or oxygen saturation was not measured at diagnosis but in TB SeWA the data are available
Measurement error
If there is a significant difference in mean weight and/or mean BMI between the audit team and TB SeWA

TB SeWA = Severe TB Web Application; Ni-kshay = web-based information management system of TB programme; BMI = body mass index.

Investigators extracted the routine captured triage data from TB SeWA (date of extraction 1 March 2025) for comparison with data from onsite audit observation in EpiCollect5 (merged using the unique identifier Ni-kshay ID).

### Data analysis

**FIGURE 2.**
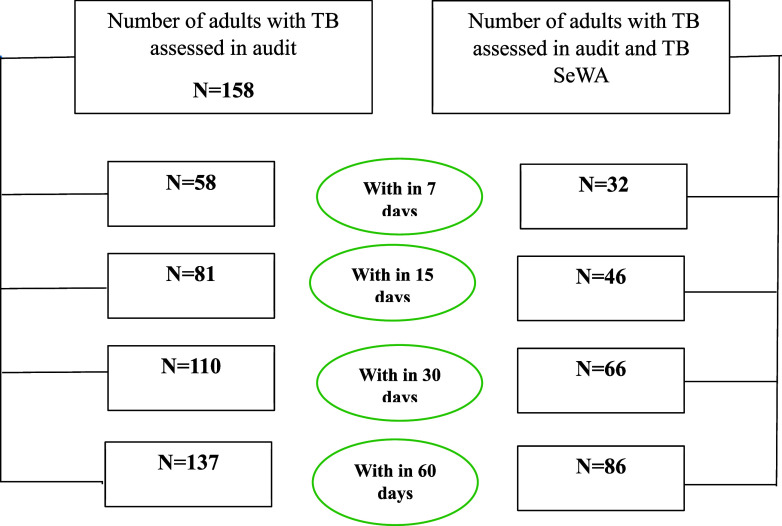
Bias and limits of agreement for paired measures of weight measured during the audit and weight extracted from TB SeWA among persons with TB in the triage audit of selected districts in Tamil Nadu, 2024–2025. wti = weight measured in kilogram by the investigating (audit) team; wttri = weight measured in kilogram at the TB notification facility at the time of diagnosis.

Analysis for the first objective was conducted using MS Excel, calculating reported and true triage coverage by quarter and district. For the second objective, STATA 14.0 was used, restricting analysis to patients with triage data in TB SeWA. Classification error was assessed using McNemar’s χ^2^ test, while reporting error was measured through proportions. Agreement on triage indicators between audit findings and TB SeWA was evaluated using kappa statistics (Table 3). Measurement error in weight and BMI (severe: <16 kg/m^2^; very severe: <14 kg/m^2^) was quantified using Bland-Altman plots and intraclass correlation (Figure 2). Prevalence- and bias-adjusted kappa statistics were applied when expected agreement exceeded observed values. Significant weight and BMI changes were not expected within the first 2 weeks for triage-positive patients. Operational definitions are summarised in Table 2.

**TABLE 3.** Comparison of various variables and triage-positive between data captured in TB SeWA and by the triage audit team in 11 districts of Tamil Nadu, 2024–2025 (*N* = 98).

Variable	TB SeWA	Triage audit	P value^a^	Agreement kappa
BMI < 14 kg/m^2^	7 (7.1)	16^b^ (17.6)	0.003	0.40
BMI < 16 kg/m^2^	29 (29.6)	27^b^ (29.7)	0.28	0.61
Pedal oedema	2 (2)	13 (13.3)	0.002	0.1
Oxygen saturation < 94%	3 (3.1)	11 (11.2)	0.03	0.7^c^
Not standing without support	6 (6.1)	12 (12.2)	0.11	0.15
Triage-positive	27 (27.6)	35 (35.7)	0.19	0.11

a McNemar χ^2^ test.

b Number restricted to 91 as, at the time of audit, 7 adults with TB could not stand to measure anthropometry.

c Prevalence- and bias-adjusted kappa.

### Ethics

Ethics approval was provided by the Institute Human Ethics Committee of ICMR-NIE, Chennai. A waiver of consent was obtained for secondary data usage, while written informed consent was required for primary data collection in the triage audit. All necessary administrative approvals were secured before the study commenced.

## RESULTS

**FIGURE 3.**
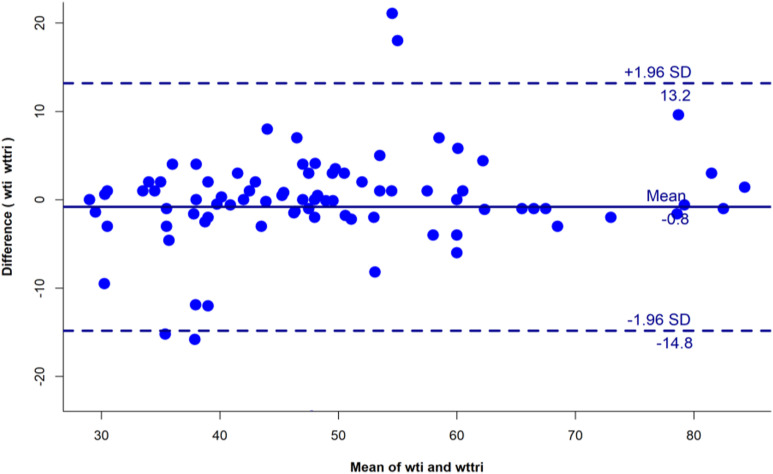
Distribution of study participants enrolled in the triage audit by time since notification in selected districts of Tamil Nadu, India, 2024–2025.

A total of 48,905 adults with drug-sensitive TB were notified, and 55,441 were triaged and entered in TB SeWA. The reported triage coverage based on aggregate numbers was 113.4%. This varied from 102% to 152.7% by quarters and 96% to 163% by districts. Of the 48,905 patients notified, 41,194 (84.2%) were traceable in TB SeWA. This true triage coverage varied from 81.7% to 89.7% by quarters and 74.4% to 93.64% by districts. In 5 of 11 districts, TB SeWA-reported triage coverage was more than 100%, while actual coverage based on individual linkage to Ni-kshay was 60%–90% (Figure 3). TN-KET, Tamil Nadu’s differentiated TB care model, targets only drug-sensitive TB patients from the public sector, whereas the TB SeWA platform allows entries also for drug-resistant and privately notified patients.

### Quality of triage

A total of 158 adults with TB (mean age: 49 years, SD: 14.7; 65% men) underwent the triage audit. More than half (54.4%) of the adults were notified by tertiary care facilities. The triage audit was conducted within 7 days for 36.7% of the patients, within 14 days for 51.3%, within 30 days for 69.6%, and within 60 days for 86.7%. Data for 98 (62%) patients were available in TB SeWA for further analysis. Triage-positive rates were higher in the audit than in TB SeWA (35.7% vs. 27.6%, *P* = 0.19), with significant differences noted for pedal oedema, oxygen saturation, and performance status. Discrepancies were observed across all triage variables, with agreement (kappa) ranging from 0.1 to 0.7, consistent across time points (see Table 3 and Supplementary Table). Only 35% reported leg examination, and 98% reported oxygen saturation measurement. Of the 98 patients, 91 (92.9%) had anthropometric measurements; 7 were unable to stand. Among the 91 patients, the mean weight was 48.2 kg (audit) vs. 49.1 kg (TB SeWA), with an intraclass correlation of 0.86 and an average bias of 0.8 kg; 7.7% had differences beyond limits of agreement. The mean BMI was 18.9 kg/m^2^ (audit) vs. 19.3 kg/m^2^ (TB SeWA).

## DISCUSSION

In 11 Tamil Nadu districts that missed the 30% TB death reduction goal, key triaging issues emerged. True triage coverage based on individual tracking was 84%, much lower than the 100% coverage reported in routine reviews, a discrepancy also noted in the audit. Triage quality was suboptimal, with higher burdens of triage-positive patients, pedal oedema, low oxygen saturation, and inability to stand without support, as confirmed by audit data.

The triage audit was conducted at TB patients’ residences by an independent team, ensuring a reliable assessment. A single trained assessor cross-verified triage measures across 11 districts using a standard checklist for consistency. Initially, the audit aimed to include recently notified (within 7 days) adults with TB, but logistical challenges made this impractical within blocks. Consequently, study participants in the intensive treatment phase were included. A key audit limitation is the 60-day delay between diagnosis and field assessment in a few patients, caused by logistical constraints in scheduling domiciliary visits. This lag may affect weight, BMI, and clinical status, particularly in triage-positive individuals with undernutrition. For more than one third of the enrolled patients in the triage audit, we could not trace their status in TB SeWA (as they were not triaged), which effectively reduced our sample size. This was unavoidable. As the purpose was to identify errors committed in the triage items, which will inform the subsequent action plan for quality improvement, rather than generalising the findings, it may not be a major methodological limitation.

Limitations notwithstanding, the findings are relevant to the ongoing TN-KET. Over 2 years, the effective triage coverage for these districts was 84%, which is less than the expected target of 90% under TN-KET. Based on this true triage coverage, out of 11 districts, only one district achieved the target of 90%. Contrary to what is perceived, many adults with TB are not getting triaged, some of whom could be triage-positive. In TN-KET, extra-pulmonary TB, transfer out of districts, and diagnosis in higher-level facilities (busy clinics) have been identified as predictors of not undergoing triaging.^9,14^ New health staff in NTEP and health systems may not be aware or trained. Districts not achieving the 30% TB death rate reduction should use true triage coverage based on individual-level tracking between Ni-kshay and TB SeWA and not reported triage coverage based on aggregate numbers. When it comes to triage quality, many severely ill patients are getting missed in TB diagnosis. This was seen overall as well as for each indicator contributing to triage positivity. This is due to a combination of measurement errors involving incorrect measurement and/or non-measurements (pedal oedema). This indicates the possibility of ignoring objective triage assessments of those who are at borderline risk (look apparently healthy) although TN-KET emphasises objective assessment.^14^ The possibility of failure to document in TB SeWA despite completing the triage assessment also cannot be ruled out. This is supported by the anecdotal observation that in many districts entry from a paper-based triage tool to TB SeWA happens at the end of the month and not near-real-time. Adults with TB reported that oxygen saturation was checked for most of them. The exact reasons need to be explored in each district and could be context specific. While the differences in triage-positive based on the triage audit and TB SeWA cannot be compared as both assessments were not done at the same time, we have reasons to believe that the differences are real. First, weight does not significantly change in the first 2 weeks, especially among patients with severe and very severe undernutrition.^15^ Mean weight and mean BMI were lower in the triage audit when compared with TB SeWA. Filling of paper-based triage tools happens within a median of 1 day.^9^ Hence, the data in TB SeWA is reflective of triage indicators at diagnosis. Triage audit timing varied, but regardless of when it occurred, mean weight and BMI were consistently lower than in TB SeWA.

To assess if triaging was objectively performed, the patients were asked about checks for pedal oedema and oxygen saturation. While most recalled oxygen checks, only one third of the patients confirmed leg examination. This may be due to the greater acceptance of equipment-based assessments over those requiring physical contact, possibly driven by perceived importance or fear of transmission—an area for further investigation.

Triangulating routine data with onsite assessments reveals key insights. First, real-time data entry is essential for timely audits via TB SeWA. Second, programme managers should prioritise individual-level tracking, especially when high triage coverage does not align with lower TB death rates. Though developed for India, these lessons apply to other TB and disease programmes. Third, health care provider training must stress all triage variables, backed by regular supervision. Lastly, field findings should inform action plans to complete the audit cycle.

## CONCLUSION

In districts failing to meet the 30% TB death reduction target, a clear gap exists between reported and actual triage coverage, with the latter being much lower. Triage quality was also inadequate. These districts should adopt individual-level tracking and focus on improving triage quality to ensure timely admission of triage-positive patients. Enhancing real-time data entry and supervision can help prevent misclassification of disease severity and support TB death reduction at the state level.

## Supplementary Material


