# Supplementary material for: Role of triage audit in an ongoing differentiated TB care initiative to reduce deaths in Tamil Nadu, India
Source: Public Health Action. 2025 Sep 3;15(3):118–23. doi: 10.5588/pha.25.0015 (PMC12421824; doi:10.5588/pha.25.0015)
Supplement: Supplementary file 1 [file pha25-0015_supplementarydata1.pdf]

Q5: *Please provide supplement material for this article.*

Please find below the supplementary table as requested:
Supplementary Table 3s.

Comparison of selected triage-positive variables captured via TB SeWA versus the triage audit team within one month of TB notification across 11 districts of Tamil Nadu (2024–2025) [N=66]

Variable	TB SeWA		Triage audit		P-value#	Agreement kappa
BMI <14 kg/m2	4	(6.1)	7	(11.5)	0.18	0.50
BMI<16 kg/m2	19	(28.8)	16	(26.2)	0.71	0.70
Pedal oedema	1	(1.5)	10	(15.1)	0.003	0.16
Oxygen saturation<94%	1	(1.5)	8	(12.1)	0.02	0.73**
Not standing without support	2	(3)	10	(15.1)	0.02	0.64**
Triage-positive	14	(21.2)	22	(33.3`)	0.09	0.18

McNemar chi-square test; *Number restricted to 61 as at the time of audit 5 adults with TB (assessed within one month) could not stand to measure anthropometry **Prevalence and bias adjusted kappa
